# A Comprehensive Genomic Analysis of Chinese Indigenous Ningxiang Pigs: Genomic Breed Compositions, Runs of Homozygosity, and Beyond

**DOI:** 10.3390/ijms241914550

**Published:** 2023-09-26

**Authors:** Shishu Yin, Zhi Li, Fang Yang, Haimin Guo, Qinghua Zhao, Yuebo Zhang, Yulong Yin, Xiaolin Wu, Jun He

**Affiliations:** 1 College of Animal Science and Technology, Hunan Agricultural University, Changsha 410128, China; yinshishu2019@126.com (S.Y.); zhili006@gmail.com (Z.L.); y6211829@163.com (F.Y.); gpinming@163.com (H.G.); 15334930839@163.com (Q.Z.); ybzhangfd@126.com (Y.Z.); 2 Key Laboratory for Evaluation and Utilization of Livestock and Poultry Resources (Pigs) of the Ministry of Agriculture and Rural Affairs, Changsha 410128, China; yinyulong@isa.ac.cn; 3 Animal Nutrition Genome and Germplasm Innovation Research Center, Hunan Provincial Key Laboratory for Genetic Improvement of Domestic Animal, College of Animal Science and Technology, Hunan Agricultural University, Changsha 410128, China; 4 Laboratory of Animal Nutrition Physiology and Metabolism, The Institute of Subtropical Agriculture, The Chinese Academy of Sciences, Changsha 410125, China; 5 Council on Dairy Cattle Breeding, Bowie, MD 20716, USA; 6 Department of Animal and Dairy Sciences, University of Wisconsin, Madison, WI 53706, USA

**Keywords:** crossbreeding, meat quality, genomic inbreeding, preservation, SNP, swine

## Abstract

Ningxiang pigs are a renowned indigenous pig breed in China, known for their meat quality, disease resistance, and environmental adaptability. In recent decades, consumer demand for meats from indigenous breeds has grown significantly, fueling the selection and crossbreeding of Ningxiang pigs (NXP). The latter has raised concerns about the conservation and sustainable use of Ningxiang pigs as an important genetic resource. To address these concerns, we conducted a comprehensive genomic study using 2242 geographically identified Ningxiang pigs. The estimated genomic breed composition (GBC) suggested 2077 pigs as purebred Ningxiang pigs based on a ≥94% NXP-GBC cut-off. The remaining 165 pigs were claimed to be crosses, including those between Duroc and Ningxiang pigs and between Ningxiang and Shaziling pigs, and non-Ningxiang pigs. Runs of homozygosity (ROH) were identified in the 2077 purebred Ningxiang pigs. The number and length of ROH varied between individuals, with an average of 32.14 ROH per animal and an average total length of 202.4 Mb per animal. Short ROH (1–5 Mb) was the most abundant, representing 66.5% of all ROH and 32.6% of total ROH coverage. The genomic inbreeding estimate was low (0.089) in purebred Ningxiang pigs compared to imported western pig breeds. Nine ROH islands were identified, pinpointing candidate genes and QTLs associated with economic traits of interest, such as reproduction, carcass and growth traits, lipid metabolism, and fat deposition. Further investigation of these ROH islands and candidate genes is anticipated to better understand the genomics of Ningxiang pigs.

## 1. Introduction

Ningxiang pigs are among the four most renowned Chinese indigenous swine breeds, known for their meat quality, flavor, environmental adaptability, and disease resistance. Compared to imported commercial pigs, Ningxiang pigs have better meat quality and fatty acid components, making them a preferred choice for pork products [1]. Moreover, Ningxiang pigs have exhibited remarkable resilience to the local environment and extensive feeding in Hunan, a southern province of China. As a result, pork products from Ningxiang pigs command a premium price compared to commercial crossbred pigs. However, their market potential has been limited by their slow growth rate and low feed efficiency. To enhance their retail value, intensive crossbreeding has been widely used in the past decades, which has posed a threat to their preservation over time. Thus, understanding the genomic compositions and characterizations of Ningxiang pigs is becoming increasingly crucial to preserve this local swine breed and ensure sustainable breeding and farming [2].

Knowing the genomic breed composition of Ningxiang pigs is crucial for breed registration and helps avoid unintentional crossing. Pedigree information has traditionally been used to establish purebred animal registries and estimate the ancestral breed composition of crossbred animals. However, for indigenous pigs, pedigree information can be missing or incomplete, and record errors are common. Therefore, estimating genomic breed compositions (GBC) is becoming an increasingly useful tool for identifying purebred pigs [3,4]. GBC refers to the partition of the genomes of an animal inherited from its ancestors or ancestral breeds, yet the precise interpretations are subject to how GBC is estimated [5]. The U.S. Council on Dairy Cattle Breeding (CDCB) uses an alternative term for GBC, namely Breed Base Representations (BBR), which are obtained from a genomic prediction model with the breed as the phenotypes (100 for each reference breed and 0 otherwise) [6]. Depending on the statistical methods, GBC may represent genomic percentages identical-in-state with reference breeds instead of identical-by-descend with their ancestors. For example, the admixture model postulates that an observed genotype for a progeny is an instance of a multinomial distribution, with genotype probability being a mixture governed by the allelic frequencies of the ancestors or reference populations. Hence, GBC is estimated by the weights or admixture coefficients [7]. Linear regression estimated GBC to be adjusted regression coefficients of coded genotypes for a progeny based on the ancestral allele frequencies and bounded between 0 and 1 [8,9]. A genomic prediction model estimates the SNP effects on candidate ancestry breeds as binary traits [10,11]. Recently, Wu et al. [12] proposed a causal interpretation of GBC based on path theory, which decomposed the relationships between ancestors and their progenies into direct and indirect ancestral breed (path) effects. GBC was then measured by the relative ratio of direct (D-GBC) and combined (C-GBC) breed determination, respectively, from each putative ancestry breed to a progeny.

He et al. [2] conducted a preliminary study to evaluate the effectiveness of genomic mating as a sustainable selection strategy for genetic improvement using genomic data and simulated phenotypes in 515 Ningxiang pigs. An initial and arguable estimation of inbreeding was provided as well. However, genomic characterizations of Ningxiang pigs in more significant numbers, including genomic breed compositions, precise inbreeding estimates, and possible population stratifications, have not been addressed. These are crucial pieces of information for optimal preservation and sustainable production of Ningxiang pigs. This study aimed to fill the gap by estimating GBC in a more extensive set of Ningxiang pigs and probing into their genomic characterizations based on runs of homozygosity (ROH), that is, continuous homozygous genomic segments in an individual animal due to the parents transmitting identical haplotypes to their offspring [13]. In practice, ROH information can be used to infer inbreeding history. Long ROH results from more recent inbreeding, whereas short ROH indicates sharing ancient common ancestors [14]. Moreover, the presence of genomic regions with a high frequency of ROH may also suggest the occurrence of selection for economically important traits and environmental adaptation [15,16]. Important candidate genes in ROH islands may be related to economic traits of interest, such as reproduction, carcass and growth traits, lipid metabolism, and fat deposition.

## 2. Results and Discussion

### 2.1. Characterization of Reference Populations

**Figure 1:**
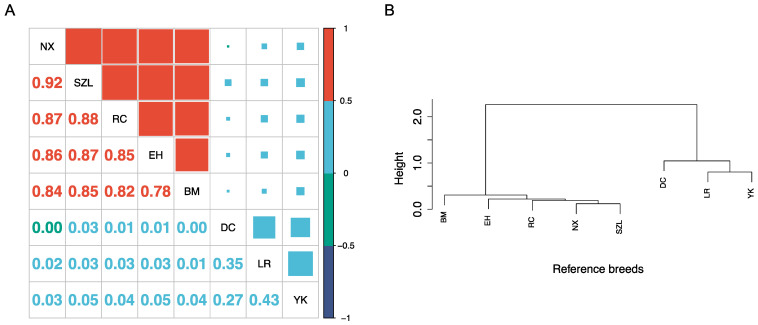
The relationships between the eight reference populations are revealed by (**A**) correlations of allele A frequencies and (**B**) hierarchical cluster dendrograms, respectively. NX: Ningxiang pig, SZL: Shaziling pig, RC: Rongchang pig, EH: Erhua pig, BM: Bamaxiang pig, DC: Duroc pig, LR: Landrace pig, YK: Yorkshire pig.

Allelic frequencies varied between these breeds (Table 1). Overall, the three imported pig breeds (Duroc, Landrace, and Yorkshire) exhibited higher allele A frequencies (0.495–0.496) than the four Chinese pig breeds (0.427–0.431). These four indigenous breeds demonstrated close correlations in allele A frequencies, ranging from 0.78 between Bama and Erhua pigs to 0.92 between Ningxiang and Shaziling (Figure 1A). In contrast, the relationships of allele A frequencies between the three imported swine breeds were relatively lower, ranging from 0.27 between Yorkshire and Duroc pigs to 0.43 between Yorkshire and Landrace pigs (Figure 1A). The correlations of allele A frequencies between the four indigenous pigs and the three imported pigs were nearly zero (i.e., 0.00–0.05) (Figure 1A). Hierarchical clustering analysis based on allele A frequencies assigned the seven reference pig populations into two primary clusters (Figure 1B). The first cluster consisted of the three imported pig breeds—Duroc, Landrace, and Yorkshire—while the second cluster comprised the four indigenous Chinese pig breeds, Bama, Ningxiang, Erhua, and Shaziling. Within the cluster of indigenous Chinese pig breeds, Ningxiang and Shaziling pigs exhibited the shortest genetic distance.

SNPs are common genetic variations reflecting single-nucleotide (A, T, C, or G) alterations in the DNA sequence. The differences in SNP allelic frequencies between breeds may have reflected their varied breeding practices, genetic drift, and selection pressures in general. Breeding practices such as selective breeding, crossbreeding, and inbreeding can influence allelic frequencies by promoting the propagation of specific alleles and reducing the frequency of others. For example, the three imported pig breeds have been under high selection for meat production. Hence, the allelic frequencies of genes associated with the traits under selection may have increased in these breeds over time. In contrast, indigenous Chinese breeds may have higher frequencies of genes (or SNPs linked to these genes) related to local environmental adaptation and disease resistance. Genetic drift happens in small populations due to chance events that lead to random fluctuations in allelic frequencies. Additionally, genetic drift can significantly impact allelic frequencies over time when a local population gets smaller. However, it should be noted that the SNP A/B allelic notation is just one of the DNA strand designations used for SNP genotypes without any implications on their frequencies, genetic functions (associated effects), or genetic inheritance in general.

These four indigenous breeds demonstrated close correlations in allele A frequencies, reflecting more relative mutual genetic similarities between them due to similar breeding practices and adaptions to the local environments. In particular, the highest correlation between Ningxiang and Shaziling pigs was due to their geographical proximity because both breeds have been domesticated and raised in neighboring areas of Hunan Province. There could be frequent gene flows between the two populations due to shared environments, natural mating, or human-mediated exchange of animals in the history of this development. In contrast, the relationships between the three imported swine breeds were relatively remote. Their correlations may stem from sharing remote common ancestries and alleles identical-in-state among western pig breeds.

Our results align with previous studies based on microsatellites and SNP markers (e.g., Megens et al.; Ai et al.) [17,18]. Megens et al. [17] suggested that the extremely low genetic similarities between Chinese and Western pigs could represent different domestication or breed formation processes. The formation of Chinese indigenous and imported pig breeds might have followed different processes of breed formation, with each group experiencing various historical events, founder effects, or population bottlenecks. These processes can lead to different genetic makeups and low genetic correlation between the groups.

Geographic isolation and divergent selection pressure can be the reasons for the differences between Chinese and Western pigs. Firstly, Chinese pig breeds have unique genetic architectures (or genetic backgrounds) shaped by their geographic isolation and long-standing breeding practices, which are very different from these western pig breeds. On the one hand, Chinese pigs have been raised in China for thousands of years, and during this time, they have developed unique genetic traits adapted to the local environment and production systems. On the other hand, there have been limited gene flows between these populations throughout history due to geography and political boundaries that prevent the exchange of livestock and genetic material with other countries. This led to a relatively closed gene pool for Chinese pig breeds, with less opportunity for genetic mixing with Western breeds before the mid-1990s. Secondly, the genetic differences between Chinese and Western pig breeds may be the result of different selection pressures and breeding goals. Chinese pig breeds have traditionally been selected for traits such as disease resistance, hardiness, and adaptability to local environments, whereas Western pig breeds have been intensively selected more for meat production and growth rate. These different selection pressures may have led to differences in the genetic architecture of these populations over time. This clustering pattern again reflects the genetic differences between the imported and indigenous breeds, highlighting their distinct genetic backgrounds and lineages. Within the cluster of indigenous Chinese pig breeds, Ningxiang and Shaziling pigs exhibited the shortest genetic distance. Hence, these two indigenous breeds have a high degree of genetic similarity, possibly due to their shared ancestry, geographical proximity, or similar selection pressures.

### 2.2. Estimation of Genomic Breed Compositions of Purebred Ningxiang Pigs

**Figure 2:**
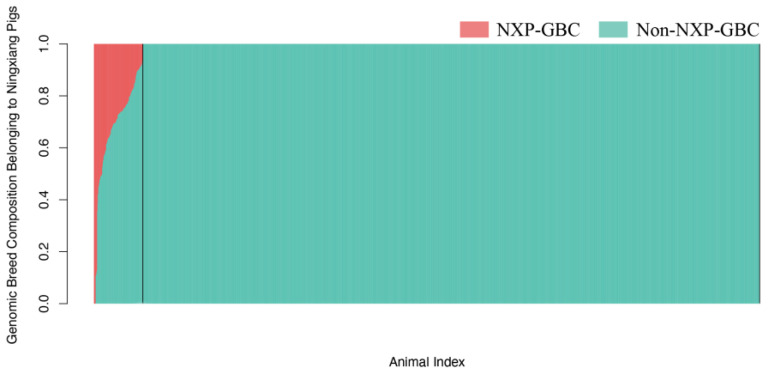
Distributions of estimated genomic breed compositions (GBC) belonging to Ningxiang pigs in 2242 experimental pigs, which were obtained using the U10K, with the identified change points represented by the vertical black lines.

In practice, identifying purebred Ningxiang pigs from crosses and other indigenous pigs is challenging due to the increasing use of crossbreeding of Ningxiang pigs with domestic and imported pigs in the past decades, stimulated by the market demand for pork products from Ningxiang pigs. Similar to other indigenous pigs, pedigree information is often unavailable for pigs in non-nucleus breeding populations, and missing pedigree records are common even in nucleus breeding populations. With high-throughput genotyping available, purebred animals can be identified based on genomic breed compositions attributable to possible breed origins. The present study estimated genomic breed compositions for the 2242 experimental pigs using four SNP panels (U1K, U5K, U10K, and 12K). Figure 2 displays the distributions of estimated genomic compositions belonging to Ningxiang pigs (NXP-GBC) for the 2242 animals in ascending order obtained with the U10K panel. The other three SNP panels gave similar patterns yet varied percentages of animals, with a 100% NXP-GBC percentage. The portion of animals with perfect (100%) NXP-GBC decreased as the panel size increased: 95.6% with U1K, 93.9% with U5K, and 92.6% with U10K and 12K. The U1K panel identified 2143 pigs with NXP-GBC = 100%, and the 12K panel identified 2077 pigs with NX-GBC = 100%. These results suggested that the stringency for identifying purebred animals increases with the SNP panel sizes. Statistically, this is equivalent to saying that the probability of false positive errors (type I errors) will increase and the probability of false negative errors (type II errors) will increase as the SNP panel size decreases. Based on this, 100% NXP-GBC cut-off, approximately 3.57–7.36% of these 2242 experimental animals were crossbred or non-Ningxiang pigs.

Determining an appropriate cut-off for identifying purebred animals is crucial and challenging, and this cut-off can vary with species and populations. As mentioned above, enforcing a 100% NX-GBC for an animal to be classified as a purebred pig is too stringent and can result in a high false negative rate, potentially misidentifying genuine purebred pigs as crossbred pigs. In this study, we also applied nonperfect cut-offs: NYP-GBC ≥ 0.94, NYP-GBC ≥ 0.90, and NYP-GBC ≥ 0.80 (Table 2). The percentage of putative purebred Ningxiang pigs remained unchanged when the NXP-GBC cut-off changed from 100% to 94%. This was because we used a filter that nullified all GBC components by less than 1% in the computation. However, without this filter, the 94% cut-off led to only a slightly higher estimated NXP-GBC on average. The portion of putative purebred animals increased to 95.6–93.3% when the cut-off dropped to NXP-GBC ≥ 0.90, and it further rose to 94.5–96.4% when the lower cut-off was taken (i.e., NYP-GBC ≥ 0.80). For example, using the U10K panel, the number of putative purebred Ningxiang pigs was 2077, 2092, and 2120 when the NXP-GBC cut-off was set at ≥0.94, ≥0.90, and ≥0.80, respectively (Table 2). With the 10K panel, the change-of-point analyses identified 93.6% NXP-GBC as a change point starting to deviate from 100% NXP-GBC. The C12K panel gave the same change point estimate as the U10K panel, but we also noted that using a smaller panel size led to a lower percentage as the change point. For example, the change point was identified at 91.6% NXP-GBC using the U5K panel results. In the present study, we used NXP-GBC ≥ 0.94 as a reasonable GBC cut-off for identifying purebred Ningxiang pigs.

Precisely speaking, the GBC estimated using an admixture model corresponds to the proportion of genomes that are identical-in-state rather than identical-by-descent. Since all pig breeds are correlated due to shared common ancestors, the admixture model tends to produce a small GBC value for a related or remote ancestry breed. The higher the genomic similarity, the more noise. Consequently, the estimated GBC for a specific breed is not always 100%. This phenomenon is referred to as “the Impure purebred Paradox” [19]. Statistically, this situation could lead to increased false-negative errors in purebred animal identification. Conversely, using a less stringent cut-off could increase false-positive errors. Our results suggested a cut-off of approximately 94% NYP-GBC, which identified 2077 animals with a cut-off as low as NYP-GBC ≥ 0.94. This cut-off coincides with the 94% BBR threshold for purebred genomic evaluation in dairy cattle in the U.S.A. [20].

### 2.3. Population Stratification and Identification of Crossbred Pigs

**Figure 3:**
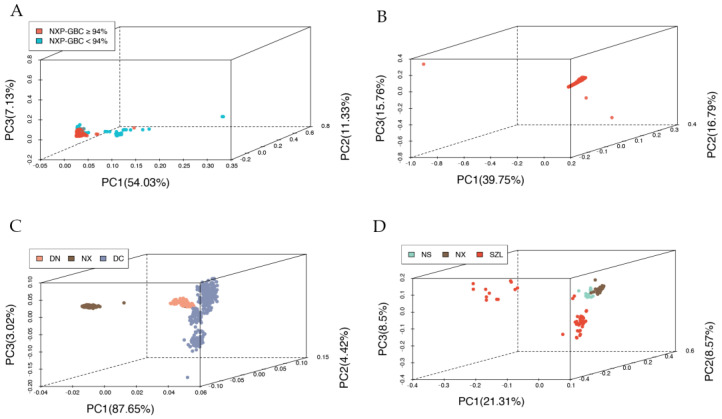
Cluster analysis using the principal components of allele A frequencies for: (**A**) 2242 Ningxiang pigs; (**B**) 2077 purebred Ningxiang pigs, the red dots represent animals with NXP-GBC ≥ 94%; (**C**) 348 Duroc pigs, 107 D×N pigs, and 400 Ningxiang pigs; (**D**) 50 purebred Ningxiang pigs, 15 crossbred N×S pigs, and 50 Shaziling pigs.

The principal component analysis (PCA)-based clustering analysis revealed clear patterns of clusters with the 2242 experimental animals (Figure 3A). The animals with NXP-GBC ≥ 94% (Figure 3A: blue dots) mostly fell into a single cluster, whereas those with NXP-GBC < 94% (Figure 3A: red dots) were scattered or presented in separate clusters. These results showed clear evidence for population stratification in geologically alleged Ningxiang pigs due to the mixture of crossbred and non-Ningxiang pigs. However, such a population mixture was not observed in the PCA-based clustering results of the 2077 putative purebred Ningxiang pigs with NXP-GBC ≥ 94%, despite the presence of outliers (Figure 3B). The 165 animals with NXP-GBC < 94% are considered to be crossbred pigs and non-Ningxiang pigs, but keep in mind that the number of crossbred and non-Ningxiang pigs can vary with SNP panel size.

**Figure 4:**
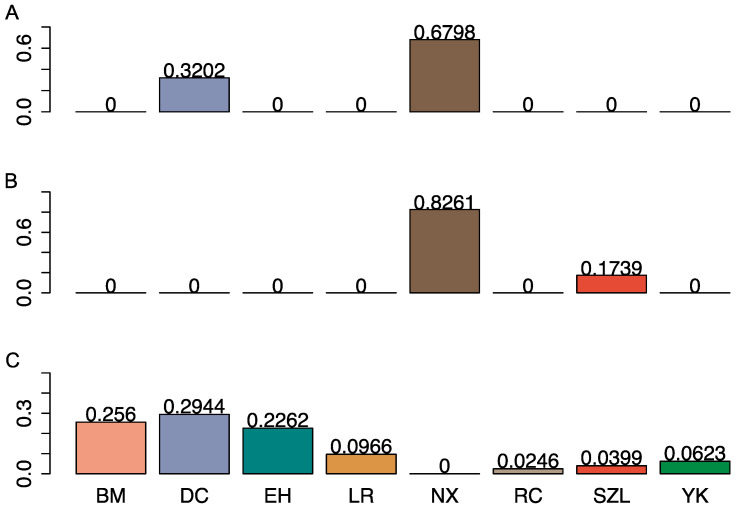
Distribution of average genomic breed compositions (GBC) pertaining to eight reference breeds: (**A**) 107 Ningxiang-Duroc (D×N) crosses; (**B**) 15 Ningxiang-Shaziling (N×S) crosses; (**C**) 6 crosses without significant genomic contributions from Ningxiang pigs. Eight colors represent eight breeds in (**A**) to (**C**).

There were 98 crossbred animals known as crossbreeding decedents between Ningxiang pigs and Duroc pigs (D×N) (Table 3). From them, we identified 67 D×N crosses with U1K and 96 D×N crosses using the U10K and C12K panels, respectively. Hence, contrary to the identification of purebred pigs, a larger panel (U10K or C12K) identified more crosses than a smaller panel (U1K or U5K). In the 2242 experimental pigs, we identified 107 D×N crosses, which included the 98 known D×N crosses. To determine if a pig was a D×N cross, we nullified all the GBC components < 5% for specific breeds and then retained only the animal with genomic contributions from Duroc and Ningxiang pigs. After scaling the GBC to 100% genomic contributions between Duroc and Ningxiang pigs, these D×N crosses had approximately 16–62% Duroc lineage and 38–84% Ningxiang lineage. On average, the 107 D×N crosses had an average of 32.0% Duroc and 68.0% Ningxiang pig genomic lineages (Figure 4A). These pigs were a mixture of mainly F1, F2, and backcross progenies of Duroc and Ningxiang pigs. By the same method, 15 crosses between Ningxiang and Shaziling pigs (N×S) were identified, which had, on average, 82.6% Ningxiang pig and 17.4% Shaziling pig genomic lineage (Figure 4B). Still, six animals had their estimated NXP-GBC approximately equaling zero (Figure 4C). These animals are not progenies from Ningxiang pigs.

PCA-based clustering plots were created for the 107 crosses together with 400 randomly selected reference Ningxiang and 348 reference Duroc pigs (Figure 3C), and for the 15 Ningxiang-Shaziling pigs together with 50 randomly selected reference Ningxiang and Shaziling pigs, respectively (Figure 3D). The results showed that these D×N crosses were genetically between the two parental breeds yet closer to Ningxiang pigs (Figure 3C). Similarly, Ningxiang pigs also had more significant contributions to the N×S crosses (Figure 3D), possibly featuring advanced backcrossing to Ningxiang pigs compared to the D×N crosses. We also noticed substantial genetic diversity or possible population stratifications in Shaziling pigs, as they were not closely clustered (Figure 3D).

### 2.4. Genomic Characterization and Runs of Homozygosity

**Figure 5:**
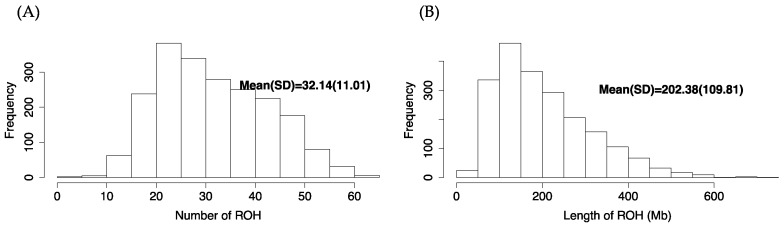
Distribution of the number and length of runs of homozygosity (ROH) in 2077 Ningxiang pigs: (**A**) average number of ROH per animal; (**B**) average length of ROH per animal.

Runs of homozygosity were identified in 2077 purebred Ningxiang pigs, which can provide information on their genetic diversity, inbreeding, and population structure. They are also useful for identifying regions of the genome subject to recent selective pressure and individuals at risk for genetic disorders or reduced fitness due to inbreeding. In total, we identified 66,746 ROH in 2077 Ningxiang pigs. The number of ROH per animal varied from 0 to 63, with an average of 32.14 ROH per animal (Figure 5A). The total length of ROH per animal ranged from 0 Mb to 706.3 Mb, with an average total length of 202.4 Mb per animal (Figure 5B).

**Figure 6:**
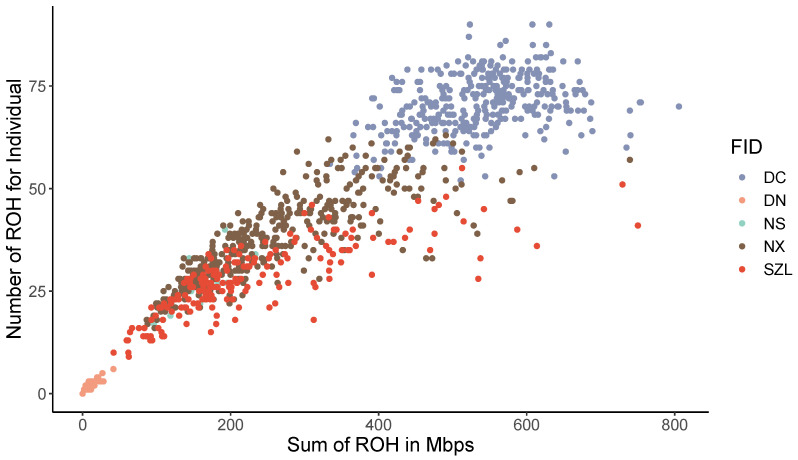
Number of runs of homozygosity (ROH) per animal versus total ROH length (Mb) per animal, evaluated in purebred and crossbred pigs.

Overall, animals with more ROH also had a higher total ROH length and, hence, more genomic coverage by these ROH (Figure 6). The two indigenous pig breeds, Ningxiang and Shaziling pigs, had an almost comparable total number (and length) of ROH. Still, the imported pig breeds (e.g., Duroc) had a significantly larger total number (and length) of ROH than Ningxiang pigs (Figure 6). This was because Ningxiang pigs are genetically closer to Shaziling pigs than Duroc pigs. The higher number of ROH in Duroc pigs (24,573) than the two Chinese indigenous pig breeds, Ningxiang (15,120) and Shaziling (5327), possibly suggests more intensive selection operated in the former in the past decades. ROH was also detected in the crossbred pigs. The presence of ROH in crossbred pigs was possibly due to haplotype sharing between parental breeds, yet the total length (number) of ROH in crossbred pigs was far shorter (less) than that in purebred pigs (Figure 6). Our results coincided with previous studies [21,22,23].

It is worth mentioning that the number and length of ROH can vary between studies in pigs (or any other species) for several reasons. Hence, they may not be directly comparable. For example, pig populations with distinct genetic backgrounds may have experienced different demographic events, selection pressures, or breeding practices, which can influence the number and length of ROH. Even within the same population, the number and length of ROH can vary between individuals due to random genetic drift or the stochastic nature of recombination events. Studies using different genotyping platforms or varying marker densities might produce different ROH results. Differences in ROH detection methods and thresholds, such as minimum length, the number of consecutive homozygous markers, and allowed heterozygous SNPs within an ROH, can lead to variations in the number and length of detected ROH. Sample sizes can also influence the statistical power and ability to detect rare or population-specific ROH.

The ROH identified in the 2077 purebred Ningxiang pigs was classified into five groups of varied length: ≤5 Mb, 5–10 Mb, 10–20 Mb, 20–40 Mb, and >40 Mb (Table 4). Overall, the abundance of ROH in these groups decreased as the average ROH length increased. The most abundant group included ROH with a size no greater than 5 Mb, representing 66.5% of all ROH and 32.6% of total ROH coverage. The average ROH in this group was 3.1 Mb per animal. The second most abundant group consisted of 21.7% of all ROH, each with an ROH length between 5 and 10 Mb, with an average ROH length of 6.7 Mb per animal. ROH in this group made up 23.1% of the total ROH coverage. The remaining three groups constituted approximately 12% of all ROH, making up approximately 45% of the total ROH coverage. The average ROH length was 14.0 Mb, 27.8 Mb, and 61.8 Mb, respectively. Although ROH segments with a length of less than 5 Mb were the most abundant, 700 animals had extra-long ROH (>40 Mb). The excess of long ROH indicated recent inbreeding events, possibly due to mating between relatives, intensive selection conducted in recent generations, and possible population bottleneck. Short ROH results from ancient inbreeding, whereas long ROH indicates recent inbreeding. Fisher [24] demonstrated that the length of an autozygous chromosome segment follows an exponential distribution with a mean equaling 100/2 g·cM, where g is the number of generations since the common ancestor. For simplicity, we let 1 cM = 1 Mb. Then, it is estimated that ROH having the average lengths in the five categories could come from a common ancestor occurring approximately 16, 8, 4, 2, and 1 generation, respectively.

Jiang et al. [25] used the same five ROH categories as ours, and they reported similar ROH frequencies in Aihui indigenous pigs: 44.7–55.1% (1–5 Mb), 28.2–37.1% (5–10 Mb), 12.1–16.6% (10–20 Mb), 2.9–5.6% (20–40 Mb), and 0.1–0.7% (>40 Mb). Nevertheless, our results demonstrated a slightly higher frequency for the first category (1–5 Mb) and a lower frequency in the last category (>40 Mb). These differences could be attributable to different indigenous breeds, possibly implying more recent inbreeding in Ningxiang pigs. Nevertheless, Jiang assessed ROH using small sample sizes (N = 30), subject to sampling bias. In contrast, our estimates were based on a large sample size (N = 2077). On average, there were 32.2 ROH per animal in Ningxiang pigs, whereas Jiang et al. [25] reported between 15.0 and 44.0 ROH in Aihui indigenous pigs. Fang et al. [26] classified ROH into three length bins: 1–5 Mb, 5–10 Mb, and >10 Mb. The short ROH (1–5 Mb) are the most frequent (18.5%), and the long ROH (>10 Mb) are the fewest (5.6%). The percentage of ROH of length 5–10 Mb was 15.9%.

**Figure 7:**
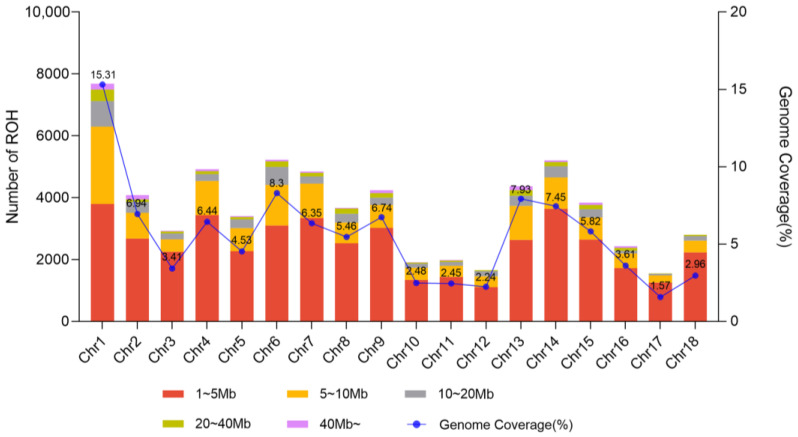
The frequency distribution of the number of runs of homozygosity (ROH) per chromosome (bar) and the percentage of each chromosome covered by ROH (blue line).

Among all the chromosomes, chromosome 1 exhibited the highest proportion (15.3%), followed by chromosome 6 (8.3%), 13 (7.9%), and 14 (7.5%) (Figure 7). Chromosome 13 had fewer ROH but a higher percentage of ROH coverage because chromosome 13 had longer ROH than chromosome 14. Conversely, chromosome 17 had the lowest proportion (1.6%) of total ROH length and a lower proportion of homozygosity. The most extended ROH block was found on chromosome 13, which spanned over 181 Mb, and comprised 2526 SNPs. On the other hand, the shortest ROH block was found on SSC3, which covered 1.00 Mb with 53 SNPs. Apparently, the intensity of ROH coverage of the genome can vary with chromosomes and with swine breeds subject to selection, genetic drifting, and adaption processes. Different breeds or populations may have various ROH distributions. For example, Tong et al. [27] reported different ROH structures among three pig breeds (Erhualian pigs, Mi pigs, and Meishan pigs) in the region of Taihu Lake, China. By comparing five Aihui Indigenous pig breeds with five Western pig breeds, Jiang et al. [25] demonstrated that chromosome 18 had the highest chromosomal coverage by ROH (31.3%) in Piétrain pigs, and chromosome 17 had the highest chromosomal coverage by ROH in Wannanhua pigs. Varying ROH coverages on different chromosomes also suggest varying selection and genetic drift operating on these chromosomes.

### 2.5. Identification of ROH Islands, Candidate Genes, and QTL

ROH hotspots, also referred to as ROH islands, are genomic regions that exhibit a higher frequency of ROH compared to other regions in the genome. These hotspots can arise for various reasons, such as reduced recombination rates, selection pressure, population bottlenecks, and inbreeding [28,29]. Some genomic regions have lower recombination rates, meaning genetic material is less likely to be exchanged between parental chromosomes during meiosis. This can lead to longer and more frequent runs of homozygosity in these regions. Additionally, SNP or marker density, genomic coverage, and other detection conditions can affect ROH distribution [30]. Positive selection can result in ROH hotspots when a beneficial allele is maintained in a population, causing the region around it to have increased levels of homozygosity. This is known as a “selective sweep”. Events such as population bottlenecks, founder effects, or migration can influence the distribution of ROH hotspots in a population [28,29]. Higher levels of inbreeding can lead to longer and more frequent ROH, as individuals are more likely to inherit identical DNA segments from both parents. Hence, genomic regions with a high frequency of ROH can harbor genes with significant implications for the events mentioned above. Szmatoła et al. [31] reported dissimilarities in the distribution of ROH islands between selected and unselected pig breeds, with a higher proportion of overlapping genomic regions in the selected breeds, suggesting a preference for artificial selection. Yuan et al. [32] identified some genes annotated within high-frequency ROH regions that were associated with disease resistance and fat deposition traits in Tongcheng (TC) pigs. Wu et al. [15] analyzed ROH islands and the result of the selection signature in the Diannan small-ear (DSE) pigs, revealing a subset of genes associated with meat quality, body size, and adaptability.

**Figure 8:**
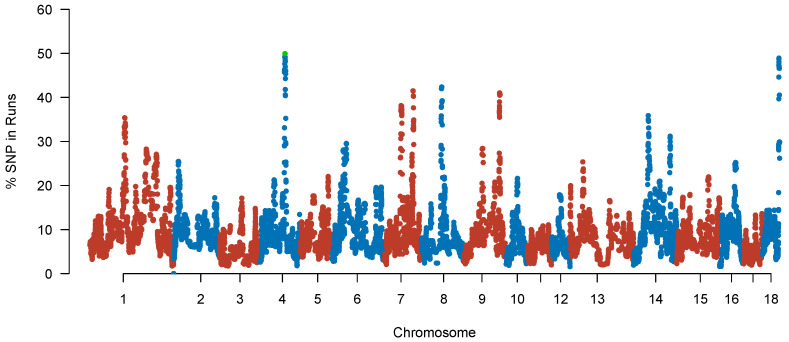
Manhattan plot of occurrences (%) of SNP in runs of homozygosity (ROH) across 2077 Ningxiang pigs. The *x*-axis represents the chromosomes, and the *y*-axis represents the SNP percentage in ROH. The dots red and blue represent different autosomes, and the green dot represent the top frequent SNP.

Our results revealed a nonuniform frequency of different SNPs within ROH regions, revealing the presence of ROH islands. Figure 8 shows the distribution of SNPs within ROH regions across the genome and the percentage of SNPs in ROH against their respective positions along the chromosome. We selected the top 1% of SNPs (29.56%) observed in an ROH in a specific population [33]. Possibly, some genomic regions may have experienced more substantial selective pressure, leading to an accumulation of homozygous regions in these regions and, therefore, the nonuniform distribution of SNPs within ROH regions across the genome. This observation is consistent with previous studies, showing that ROH regions are enriched in genes involved in important economic traits, such as growth rate, feed efficiency, and reproduction [34]. For example, the most frequent SNP in ROH (1037 occurrences, 49.93%) was located at 80.80 Mb on SSC4, with the closest gene being *NTMT2* (*METTL11B*). This gene is associated with feed conversion efficiency in pigs [35]. Nine common ROH regions were identified in these purebred Ningxiang pigs (Table 5), with 244 genes located in these ROH islands (see Table S1 for gene names). SSC7 had the most extended ROH island (4.78 Mb), which contained 54 SNPs and 72 genes, and SSC14 had the shortest island (0.48 Mb), with no gene detected. Some studies identified many genes or QTLs within SSC7 that were associated with reproduction, meat quality, and carcass traits [34,36,37].

In this study, several genes located within ROH islands have been implicated in cancer cell phenotypes. For instance, the *CCNB2* and *METTL18* genes have been identified as potential prognostic biomarkers in hepatocellular carcinoma [38,39], while the *POLG* gene has shown associations with breast cancer [40]. Furthermore, several of these genes have been linked to various aspects of animal physiology, including reproduction, growth and carcass traits, lipid metabolism, and fat deposition progress (Table 6).

However, it is essential to note that most of these findings are derived from research conducted in humans and model animals (such as mice and rats) concerning lipid metabolism and fat deposition. The Ningxiang pigs, classified as an obese-type breed, are renowned for their abundant fat content and excellent meat quality. In this study, we identified genes *ZNF280D* [41], *PLIN1* [42], *PAPPA2* [43], *SLC5A4* [44], and *CABIN1* [45], which were found to be associated with BFT in pigs, and *CCPG1* [46], *ATP1B1* [47], and *CABS1* [16], which demonstrated associations with reproductive performance in boars. Additionally, we identified some genes that simultaneously influence multiple traits. For example, the *CEBPD* gene has been reported to affect both body parameters [48] and adipogenesis [49]. Watanabe et al. [50] highlighted that pleiotropic genes are active in multiple tissues and participate in numerous biological functions within the organism.

**Table 6:** Previous studies on nine runs of homozygosity (ROH) island annotation genes in 2077 Ningxiang pigs.

Gene	Phenotype	Species	Sources
**Reproduction Trait**			
*SCAMP2*, *DPF3*, *SULT1E1*	Teat numbers	Pig	Lee et al. [51], Li et al. [52], Jiang et al. [25]
*ALDH1A2*, *TCF12*	Litter traits	Pig	Tao et al. [53], Wu et al. [54]
*NEDD4*	Sperm storage capacity	Human	Chu et al. [55]
*CCPG1*	Unilateral cryptorchidism	Pig	Da Silve et al. [46]
*SPIDR*	Primary ovarian insufficiency	Human	Heddar et al. [56]
*ATP1B1*	Sperm motility	Boar	Mańkowska et al. [47]
*ADCY10*	Sperm quality	Boar, Human	Tate et al. [57], Akbari et al. [58]
*CYP11A1*	Polycystic ovary Syndrome (PCOS) and Infertility	Human	Heidarzadehpilehrood et al. [59]
*CABS1*	Sperm structure	Pig	Zhang et al. [16]
*JCHAIN*	Polycystic ovary syndrome	Human	Zou et al.[60]
*EIF4ENIF1*	Premature ovarian insufficiency	Human	Zhao et al. [61]
*ABHD2*	Sperm activation	Human	Bononi et al. [62]
**Carcass and Growth Traits**			
*ZNF280D* *PLIN1*, *PAPPA2*, *SLC5A4*, *CABIN1*	Backfat thickness	Pig	Lee et al. [41], Gandolfi et al. [42], Wang et al. [43], Fowler et al. [44], Zhao et al. [45]
*POU6F2*	Intern organ weight	Pig	Li et al. [63]
*ARID3B*	Body fat thickness	Pig	Lee et al. [64]
**Lipid and Energy Metabolism, and Fat Deposition**			
*ZNF70*	Hepatic steatosis	Pig	Watanabe et al. [65]
*AQP9*	Fat deposition	Pig	Kuriyama et al. [66]
*PYGO1*	Steatosis	Human	Anstee et al. [67]
*CEBPD*	Adipogenesis	Pig	Óvilo et al. [49]
*POUF21*	Adipocyte differentiation	Human	Currie et al. [68]
*CREG1*	Intramuscular fatty acid content and composition	Pig	Puig-Oliveras et al. [69]
*CSK*	Browning of white adipose tissue (obesity)	Human	Huang et al. [70]
*SFI1*	Obesity	Human	Goutzelas et al. [71]
*YTHDC1*	Adipose tissue depot	Human	Rønningen et al. [72]
*RGS6*	Fat distribution	Human	Norris et al. [73]
*MMP11*	Fat accumulation	Human	Rockstroh et al. [74]
*CHCHD10*	Adipocytes metabolism	Human	Ding et al. [75]
*PTPN9*	Glucose uptake	Human	Kim et al. [76]
**Skeletal Development**			
*AMBN*	Skeletal development	Human	Kawasaki et al. [77]
*PISD*	Skeletal dysplasia	human	Zhao et al. [78]
**Immunity**			
*CD247*	T-cell receptor zeta	Pig	Van Goor et al. [79]
*MIF*	Inflammation	Bama Minipig	Li et al. [80]

By utilizing the QTL online database, we identified 901 reported QTLs within these ROH regions. Among these, 55.39% were explicitly associated with meat and carcass traits, 12.65% were related to production, 11.10% pertained to health, 10.43% were linked to reproduction traits, and 10.43% were associated with exterior traits. Notably, a significant proportion of these QTLs, 32.74% to be precise, were located in SSC7, encompassing all the traits mentioned above. Additionally, 44.81% of the QTLs discovered were explicitly associated with meat and carcass traits. We further analyzed the QTLs with known gene symbol names and presented the findings in Table S2. This study identified QTLs that were mainly associated with backfat thickness (BFT), the number of ribs, the pH of the *longissimus dorsi* (LD), and meat color (redness, L*). Regarding reproduction traits, we identified two genes associated with the teat number [52,81]. Furthermore, the number of muscle fibers [82] and the monounsaturated fatty acid-to-polyunsaturated fatty acid ratio [83] were found to be linked to meat quality traits. The presence of these genes within the ROH islands aligns with the exceptional meat quality traits and fatty acid content observed in Ningxiang pigs.

### 2.6. Estimating Genomic Inbreeding Coefficient

Finally, ROH-based genomic inbreeding coefficients, denoted by F_ROH_, were estimated for the 2077 putative purebred Ningxiang pigs, compared to other Chinese indigenous breeds and the three imported pig breeds. ROH-based genomic inbreeding offers a powerful tool for quantifying the level of inbreeding and helps identify individuals with high levels of inbreeding that may be at risk for reduced fitness or increased susceptibility to disease. ROHs are defined as contiguous stretches of homozygous genotypes along a chromosome. Hence, ROH-based genomic inbreeding indicates shared ancestry between two parental chromosomes. In this sense, genomic inbreeding based on ROH can provide a measure of direct, realized inbreeding for individual pigs, which can be more than pedigree-based methods. Pedigree-based methods have long been used to estimate inbreeding, which crucially relies on the accuracy and completeness of genealogical records. In practice, however, pedigree records can be erroneous, incomplete, or missing. Instead, ROH-based genomic inbreeding can provide more accurate estimates of inbreeding with higher resolution than pedigree-based methods. It can also allow for the detection of recent and more severe inbreeding events.

**Figure 9:**
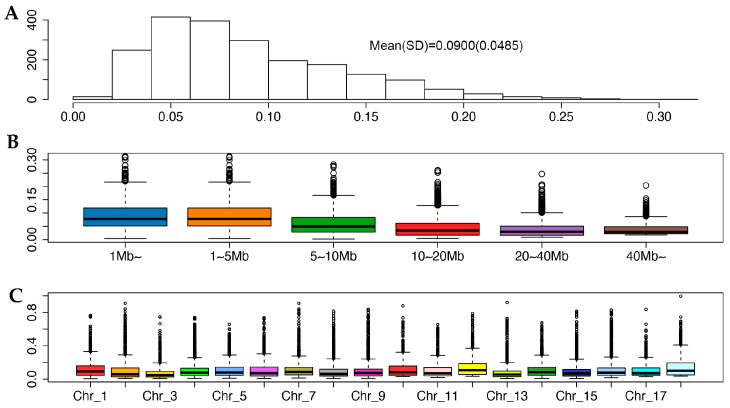
Genomic inbreeding coefficient (F_ROH_) across 2077 animals: (**A**) genome-wide; (**B**) different ROH length classes, six colors represent six kinds of ROH regions; (**C**) 18 chromosomes, the 18 bars represent 18 autosomes, and the circles represent discrete points.

The average ROH-based genomic inbreeding of the 2077 purebred Ningxiang pigs was 0.090, with a 95% confidence interval between 0.087 and 0.092 in these 2077 purebred pigs (Figure 9A). We noted that genomic inbreeding varied with the length of ROH used to compute F_ROH_, decreasing as the ROH increased (Figure 9B).

Average genomic inbreeding coefficients varied with the SNP panels used, which were 0.090, 0.060, 0.045, 0.039, and 0.039, respectively, when computed using ROH in 1–5 Mb, 5–10 Mb, 10–20 Mb, 20–40 Mb, and >40 Mb. The overall ROH-based genomic inbreeding in the 2077 putative purebred Ningxiang pigs was closer to the genomic inbreeding based on the ROH of 1–5 Mb in length, because this is the dominate category of ROH. This finding aligned with previous studies (e.g., Schiavo et al. [84]). Longer ROH is more informative for detecting recent and more severe inbreeding events, while shorter ROH is more prevalent and may represent ancient and less severe inbreeding events. Genomic inbreeding also varied considerably by chromosomes. Chromosome 3 had the lowest average F_ROH_ (0.07), whereas chromosome 18 had the largest F_ROH_ (0.15). Chromosomes 10, 12, and 16 also had more significant ROH-based inbreeding (0.12–0.14) (Figure 9C). For the majority of the chromosomes, however, they had an average ROH-based inbreeding between 0.08 and 0.11. Varied inbreeding by chromosomes was due to differences in recombination rates and patterns across the genome. Chromosomes with lower recombination rates are more likely to accumulate longer and more frequent ROH, leading to higher levels of genomic inbreeding. Differences in selection pressure, population history, and genetic drift can also contribute to the variation in genomic inbreeding by chromosomes. Certain genome regions may have experienced more intense selective pressure, leading to an accumulation of homozygous regions and higher levels of genomic inbreeding. In contrast, regions of the genome with higher genetic diversity and lower levels of selection pressure may have lower levels of genomic inbreeding.

He et al. [2] reported considerably higher genomic inbreeding (0.24–0.25) for 505 Ningxiang pigs compared to the genomic inbreed estimate in the present study. There were probably two main reasons for this discrepancy. Firstly, estimated genomic inbreeding can be substantially affected by the parameters used to define ROH [85]. In this study, we defined the minimum SNPs required for an ROH and the window width of the genomic scan that was almost twice as many as those used by He et al. [2]. The fact is that using smaller minimum SNPs and a narrow width to define an ROH tends to provide significantly higher genomic inbreeding. Another yet more important reason is that the 515 Ningxiang pigs used by He et al. [2] were taken from the nucleus preservation farmers of Ningxiang pigs. These experimental pigs used in this study had a more comprehensive geographical representation of Ningxiang pigs, including nucleus farms, non-nucleus farms, and commercial farms. It is possible that the 515 pigs collected from the nucleus breeding farm had high inbreeding due to population bottlenecking effects that happened in the past decades when small-sized nucleus breeding was practiced [86]. Population bottlenecking occurs when a population undergoes a drastic reduction in size due to political or financial reasons (e.g., lack of funding in the 20th century) and disease outbreaks. As a result of this reduction in population size, surviving individuals may have a limited set of genetic variations, which can result in an increase in homozygosity and the loss of rare alleles. Hence, genetic diversity within the population is reduced. Given the larger samples and broader representation of Ningxiang pigs, we hold that the ROH-based genomic inbreeding estimated in this study is more reliable and more representative of the inbreeding level of Ningxiang pigs.

On average, the three imported pig breeds had almost double the genomic inbreeding of purebred Ningxiang pigs, ranging from 0.18 (Yorkshire) to 0.20 (Duroc and Landrace). In contrast, the Chinese indigenous pigs had roughly comparable genomic inbreeding. For example, the average genomic inbreeding in Shaziling pigs was 0.1, slightly above the average genomic inbreeding of the purebred Ningxiang pigs. Mo et al. [87] reported lower genomic inbreeding (0.07) in Bamaxiang pigs. Jiang et al. [25] documented between 0.06 and 0.19 ROH-based genomic inbreeding in Aihui indigenous pigs. Fang et al. [26] reported an average genomic inbreeding of 0.13 in Laiwu pigs. Average genomic inbreeding in purebred Ningxiang pigs aligned roughly with reported genomic inbreeding calculated for other indigenous Chinese pigs.

Chinese indigenous pig breeds tend to have lower inbreeding than western pig breeds due to the different breeding practices and farming systems used for Chinese indigenous pig breeds and Western pig breeds. Chinese indigenous pig breeds have generally been subjected to traditional breeding practices emphasizing maintaining genetic diversity within a population. These practices have been shaped by cultural and economic factors in China and the unique environmental conditions in which the breeds have been raised. Furthermore, Chinese indigenous pig breeds have typically been raised in smallholder farming systems, where farmers have limited access to external genetic resources and rely on natural breeding methods. This can help to maintain genetic diversity within a population. In contrast, Western pig breeds have been subject to more intensive breeding programs focusing on selecting specific traits, such as fast growth, lean meat, and disease resistance. These breeding programs have often used a limited number of highly productive individuals as breeding stock, which can lead to a reduction in genetic diversity and an increase in inbreeding. However, note that certain Chinese indigenous pig breeds can have high inbreeding. For example, Zhao et al. [88] reported a 0.16 average genomic inbreeding in Meishan pigs, primarily raised in the Sichuan Province, China, and known for their high prolificacy and maternal traits. Meishan pigs have been subjected to intense selection for these traits, which has led to a high level of genomic inbreeding.

## 3. Materials and Methods

### 3.1. Animals and Genotypes

#### 3.1.1. Genotyping

The DNA samples of the experimental animals were isolated from ear and meat samples collected from the Ningxiang Chu Weixiang Slaughterhouse and Meat Processing, LLC in Hunan Province, China, and genotyped by the GeneSeek Genomic Profiling (GGP) version 2 porcine 50K SNP chips containing 50,697 SNP loci. The reference populations used to estimate GBC included three imported pig breeds (Duroc, Landrace, and Yorkshire) and five indigenous Chinese pig breeds (Bama, Erhua, Rongchang, Ningxiang, and Shaziling) (Table 1). The reference animals were genotyped by three commercial chips, namely the GGP (version 2) 50K porcine SNP chip (50,697 SNPs), the Illumina porcine SNP60 (61,565 SNPs), and the Zhongxin-1 50K porcine SNP chip (51,315 SNPs), respectively.

#### 3.1.2. Constructing SNP Panel, Genotype Imputation, and Quality Control

Data quality control for reference animals followed the protocol described by He et al. [86], where individual animals with a missing data rate > 5% were removed. The data cleaning retained 2144 experimental animals and 98 known crosses between Duroc and Ningxiang (DN) pigs. In appearance, the 98 DN pigs have all-black hair and a straight abdomen, while the purebred Ningxiang pigs have a black and white color with a drooping abdomen. These experimental animals were geologically alleged Ningxiang pigs, including purebred and crossbred Ningxiang pigs and even non-Ningxiang pigs. SNPs without map positions and those on sex chromosomes were excluded. This left 11,703 SNPs (referred to as the C12K SNP panel) for use in the study. Three additional panels were derived from the C12K SNP panels, consisting of 10,031 (U10K), 5007 (U5K), and 1013 (U1K) uniformly distributed SNPs, respectively. The SNP selection was conducted using the unified MOLO method proposed by Wu et al. [12].

For ROH detection, missing genotypes were imputed using Beagle v5.4 [89]. Genotype quality control was conducted using PLINK v1.9 [85]. SNPs were deleted based on the following filtering criteria: (1) SNP call rate < 0.95; (2) individual call rate < 0.9; (3) Hardy–Weinberg equilibrium (HWE) testing < 10^–6^. Following Meyermans et al. [29], we did not perform MAF pruning before analysis to avoid ROH loss. Finally, the remaining 44,057 SNPs were used in the following analyses: A summary of the reference populations and SNPs used in the present studies is shown in Table 1.

### 3.2. Statistical Methods

#### 3.2.1. Admixture Model

The admixture model estimated GBC by the weights of an underlying admixture distribution that governed the realization of genotypes for each animal [7]. Let there be *M* SNPs genotyped in *T* reference breeds. Assume that the allelic frequencies of these SNPs are known for these reference populations. Denote *x_jk_* as the frequency of allele A for the *k*-th SNP in the *j*-th reference population. Then, weighed allele frequency of the *k*-th SNP across the *T* reference populations was $f_{k}=\overset{T}{\underset{j=1}{\sum }}w_{j}x_{jk}$, where *ω_j_* was the weight for the *j*-reference population. Assuming Hardy–Weinberg equilibrium at each SNP, the probability of observing a genotype, denoted by *g_k_*, is the following:

(1) $$P_{r}(g_{k}|f_{k})=\{\begin{array}{c} {\left(1-f_{k}\right)}^{2},\;if\;g_{k}=0\\ 2f_{k}\left(1-f_{k}\right),\;if\;g_{k}=1\\ f_{k}^{2},\;if\;g_{k}=2 \end{array}$$

Assuming mutual independence between the *M* SNPs, the log-likelihood (denoted by *l*) computed for *M* SNPs genotyped on this animal is defined as follows:

(2) $$l=\sum_{k=1}^{M}ln(P_{r}(g_{k}|f_{k}))\;=[\sum_{k=1}^{M}g_{k}ln\left(f_{k}\right)+\left(2-g_{k}\right)ln\left(1-f_{k}\right)]+C$$

where $C=\overset{M}{\underset{k=1}{\sum }}ln\left(\begin{array}{c} 2\\ g_{k} \end{array}\right)$. Admixture coefficients, $w=\left(w_{1}\ldots \;w_{T}\right)’$, were obtained using the Broyden-Fletcher-Goldfarb-Shanno (BFGS) method under the restrictions that $w_{k}\geq 0$ and $\overset{T}{\underset{k=1}{\sum }}w_{T}=0$ [90]. BFGS is a quasi-Newton second derivative line search family method, one of the most powerful methods for solving nonlinear optimization problems. By optimizing the likelihood function of BFGS, it iteratively removed the nonzero mixing coefficient, which did not significantly improve the model fitting, and thus obtained a concise set of individual mixture coefficients. In the admixture model, the value of each admixture coefficient was between 0 and 1. The sum of admixture coefficients computed for each animal is always one under the assumption of 100% genetic contributions by the *T* known breeds to each animal.

#### 3.2.2. Principal Component Analysis and Hierarchical Clustering

Principal component analysis (PCA) was used to depict the population structure of Ningxiang pigs and other breeds [91]. The PCA projected high-dimensional genotypes onto only the first few principal components to obtain lower-dimensional data while preserving as much variation as possible. The first principal component was equivalently defined as a direction that maximizes the variance of the projected data, and subsequent components were defined as directions orthogonal to the previous ones that maximized the variance of the projected data. The principal components were computed by PLINK v1.9, and the visualization of PCA and the other results were generated by R (https://cran.r-project.org, accessed on 16 June 2023).

#### 3.2.3. Runs of Homozygosity and Genomic Inbreeding Coefficients

Runs of homozygosity (ROH) are continuous stretches of homozygous genotypes in an animal’s DNA, where both copies of the chromosome inherited from each parent have identical alleles (gene variants) for every genetic marker in that region. ROH was defined for each animal using PLINK v1.90. The criteria and thresholds were as follows [92]: (1) a minimum ROH length of 1 Mb; (2) a minimum density of an SNP in 100 Kb and the maximum gap between consecutive SNPs was set to 1 Mb; (3) a sliding window of 50 SNPs across the genome that moves on one SNP at a time; (4) up to one heterozygous SNP and two missing SNPs were allowed in a sliding window; and (5) at least 50 homozygous SNPs were included in an ROH.

- Genomic inbreeding coefficient

ROH-based genomic inbreeding was computed, reflecting the portion of the autosome genome covered by ROH. Following McQuillan et al. [93], genomic-estimated inbreeding coefficients for individual animals were calculated as follows:

(3) $$F_{ROH}=\frac{\sum L_{ROH}}{L_{genome}}$$

where ∑*L_ROH_* is the sum of the length of all ROH detected in an individual, and $L_{genome}$ is the total length of the genome that was used. In this study, $L_{genome}$ referred in particular to the size of autosomal genome coverage because sex chromosomes were excluded.

- ROH hotspots, gene annotation, enrichment analysis, and QTL screening

Genomic regions with a high frequency of ROH were identified based on the percentage of occurrences of SNP in ROH by counting the number of times an SNP was detected in these ROH across individuals in the purebred Ningxiang pigs. The obtained data were plotted against the chromosomal positions of SNPs. We selected the top 1% of SNPs (29.56%) observed in an ROH in a specific population [33]. Adjacent SNPs over the threshold were merged into genomic regions called ROH islands [33,94]. Gene annotation was conducted using the tools provided via the Ensembl website (http://asia.ensembl.org/biomart, accessed on July 2023). Then, we used ROH islands compared to the previously reported QTL for economic traits in pigs, using the gff file (https://www.animalgenome.org/cgi-bin/QTLdb/SS, accessed on 25 August 2023) comparisons in R.

## 4. Conclusions

The 2242 experimental Ningxiang pigs consisted of purebred, crossbred, and non-Ningxiang pigs sampled from Ningxiang pig farms in non-nucleus breeding regions. We identified 2077 animals to be purebred based on an NX-GBC ≥ 94% cut-off. The remaining included crosses between Duroc and Ningxiang pigs and between Ningxiang and Shaziling pigs, reflecting the increasing use of crossbreeding between Ningxiang pigs and Western (and other domestic) pig breeds in the past decades. We also investigated the distribution of ROH on the autosomes of Ningxiang pigs, identified nine common ROH regions, and annotated genes associated with important economic traits in these regions. Among the list of identified candidate genes and QTL, a considerable number are related to meat quality, fat content, and health traits. Further research is needed to elucidate the functional roles of the genes located within ROH regions and their interactions with other genes and environmental factors in determining important economic traits in purebred Ningxiang pigs. ROH-based genomic inbreeding coefficients were low for the 2077 purebred Ningxiang pigs (0.090). This genomic inbreeding estimate agreed approximately with the range of genomic inbreeding previously reported in other Chinese indigenous pig breeds but was significantly lower than the genomic inbreeding of Western pig breeds. The level of genomic inbreeding varied by chromosome, reflecting possible differences in selection pressure and genetic drift between chromosomes. We anticipate that the findings in this study can enhance our understanding of the genetic diversity and population structure of Ningxiang pigs and provide information for future breeding programs and conservation.

## Figures and Tables

**Table 1:** Summary of the animals and the SNP chips used in the present study.

Breed	N	SNP Chip	N	FreqA
Bama (BM)	297	Zhongxin-1	51,315	0.427
Duroc (DC)	1001	GGP porcine 50K	50,697	0.496
Erhua (EH)	52	GGP porcine 50K	51,315	0.430
Landrace (LR)	828	GGP porcine 50K	50,697	0.496
Ningxiang (NX)	384	GGP porcine 50K	50,697	0.428
Rongchang (RC)	139	Illumina porcine 60K	61,565	0.426
Shaziling (SZL)	190	GGP porcine 50K	50,697	0.431
Yorkshire (YK)	2711	GGP porcine 50K	50,697	0.495
Test animals	2242	GGP porcine 50K	50,697	0.423

N = number of animals; FreqA = allele A frequency estimated using 11,703 common SNPs.

**Table 2:** Number and percentage of putative purebred versus crossbred Ningxiang pigs based on varied cut-offs of estimated genomic breed compositions attributable to the genetic lineage of Ningxiang pigs (NXP-GBC).

SNP Panel	GBC Cut-Off	Purebred		Crossbred	
		N	%	N	%
C12K	NYP-GBC = 1	2077	92.64	165	7.36
	NYP-GBC ≥ 0.94	2077	92.64	165	7.36
	NYP-GBC ≥ 0.90	2092	93.31	150	6.69
	NYP-GBC ≥ 0.80	2119	94.51	123	5.49
U10K	NYP-GBC = 1	2077	92.64	165	7.36
	NYP-GBC ≥ 0.94	2077	92.64	165	7.36
	NYP-GBC ≥ 0.90	2092	93.31	150	6.69
	NYP-GBC ≥ 0.80	2120	94.56	122	5.44
U5K	NYP-GBC = 1	2105	93.89	137	6.11
	NYP-GBC ≥ 0.94	2105	93.89	137	6.11
	NYP-GBC ≥ 0.90	2107	93.98	135	6.02
	NYP-GBC ≥ 0.80	2137	95.32	105	4.68
U1K	NYP-GBC = 1	2143	95.58	99	4.42
	NYP-GBC ≥ 0.94	2143	95.58	99	4.42
	NYP-GBC ≥ 0.90	2143	95.58	99	4.42
	NYP-GBC ≥ 0.80	2162	96.43	80	3.57

C12K = SNP panel consisting of 11,703 common SNPs; U1K, U5K, and U10K = SNP panels consisting of 1000, 5000, and 10,000 uniformly distributed SNPs derived from the C12K SNP panel.

**Table 3:** Numbers of putative Duroc × Ningxiang (D×N) crosses based on different SNP panels.

Panel Density (SNP Number)	Identified D×N Crosses
C12K (11,703)	96
U10K (10,031)	96
U5K (5007)	95
U1K (1013)	67
Known crossbred	98

**Table 4:** Descriptive statistics of runs of homozygosity (ROH) in 2077 Ningxiang pigs.

ROH Range	N	N%	Mean_L (Mb)	TL%
1~5 Mb	44,415	66.54	3.09	32.61
5~10 Mb	14,451	21.65	6.72	23.11
10~20 Mb	4866	7.29	13.97	16.17
20~40 Mb	2004	3.00	27.79	13.25
40~ Mb	1010	1.51	61.84	14.86
All	66,746	100	6.30	100

**Table 5:** Runs of homozygosity islands (ROH) detected among 2077 Ningxiang pigs.

Chromosome	Region (Mb)	N_SNP	Length (Mb)	N_Gene
1	112.87~117.35	50	4.48	31
4	77.43~83.74	62	4.30	34
7	54.56~59.34	54	4.78	72
7	94.49~97.05	52	2.55	13
8	65.43~68.86	47	3.42	27
9	116.73~119.25	59	2.51	12
14	48.06~50.38	35	2.31	46
14	119.63~120.11	15	0.48	0
18	53.05~55.77	64	2.71	12

N_SNP = number of SNP; N_Gene = number of genes.

## Data Availability

The data presented in this study are available upon request from the corresponding author.

## Supplementary Materials

The supporting information can be downloaded at https://www.mdpi.com/article/10.3390/ijms241914550/s1.
