# Supplementary material for: A Comprehensive Genomic Analysis of Chinese Indigenous Ningxiang Pigs: Genomic Breed Compositions, Runs of Homozygosity, and Beyond
Source: Int J Mol Sci. 2023 Sep 26;24(19):14550. doi: 10.3390/ijms241914550 (PMC10572203; doi:10.3390/ijms241914550)
Supplement: Supplementary file 1 [file ijms-24-14550-s001.zip › ijms-2589656-supplementary.pdf]

Table S1. Detailed gene names in nine ROH islands among 2,077 purebred Ningxiang pigs.

CHR	Region (Mb)	Genes
1	112.87 ~ 117.35	*MYO1E, CCNB2, RNF111, SLTM, MINDY2, ADAM10, LIPC, U6, AQP9, ALDH1A2, POLR2M, MYZAP, CGNL1, TCF12, ZNF280D, MNS1, TEX9, RFX7, SNORA70, NEDD4, PRTG, PYGO1, DNAAF4, PIERCE2, CCPG1, ssc-mir-628, PIGB, PIGBOS1, RAB27A, RSL24D1, UNC13C*
4	77.43 ~ 83.74	*UBE2V2, MCM4, PRKDC, CEBPD, SPIDR, PRRX1, GORAB, NTMT2, KIFAP3, SCYL3, C1orf112, METTL18, SELL, SELP, F5, SLC19A2, CCDC181, BLZF1, NME7, ATP1B1, DPT, TBX19, SFT2D2, TIPRL, GPR161, DCAF6, MPC2, ssc-mir-9810, ADCY10, MPZL1, RCSD1, CREG1, CD247, POU2F1*
7	54.56 ~ 59.34	*MFGE8, ABHD2, RLBP1, FANCI, POLG, ssc-mir-9-3, RHCG, U6, TICRR, KIF7, PLIN1, PEX11A, WDR93, MESP1, MESP2, ANPEP, AP3S2, ARPIN, ZNF710, IDH2, SEMA4B, CIB1, GDPGP1, TTLL13, NGRN, VPS33B, PRC1, UBE2Q2, FBXO22, NRG4, TMEM266, ETFA, ISL2, SCAPER, RCN2, PSTPIP1, TSPAN3, PEAK1, HMG20A, LINGO1, ODF3L1, CSPG4, SNX33, IMP3, SNUPN, PTPN9, SIN3A, MAN2C1, NEIL1, COMMD4, C15orf39, PPCDC, SCAMP5, RPP25, COX5A, FAM219B, MPI, SCAMP2, ULK3, CPLX3, LMAN1L, CSK, CYP1A2, CYP1A1, EDC3, CLK3, ARID3B, UBL7, SEMA7A, CYP11A1, CCDC33, STRA6*
7	94.49 ~ 97.05	*PCNX1, SIPA1L1, RGS6, DPF3, ZFYVE1, RBM25, PSEN1, PAPLN, NUMB, RIOX1, ACOT6, DNAL1, PNMA1*
8	65.43 ~ 68.86	*UBA6, TMPRSS11D, TMPRSS11A, TMPRSS11F, TMPRSS11B, TMPRSS11E, U6, YTHDC1, UGT2B31, SULT1B1, SULT1E1, CSN2, ODAM, FDCSP, CSN3, CABS1, AMTN, AMBN, ENAM, JCHAIN, UTP3, RUFY3, GRSF1, SLC4A4, GC, NPFFR2, ADAMTS3*
9	116.73 ~ 119.25	*ASTN1, BRINP2, COP1, PAPPA2, RABGAP1L, CACYBP, MRPS14, TNN, KIAA0040, TNR, SCARNA3, MIR488*
14	48.06 ~ 50.38	*PATZ1, DRG1, EIF4ENIF1, SFI1, PISD, PRR14L, DEPDC5, U6, YWHAH, SLC5A1, SLC5A4, ZNF280B, PRAME, RSPH14, GNAZ, RAB36, BCR, SPECC1L, ADORA2A, UPB1, GUCD1, LRRC75B, GGT5, SUSD2, CABIN1, DDT, GSTT4, MIF, SLC2A11, DERL3, SMARCB1, MMP11, CHCHD10, C22orf15, VPREB3, ZNF70, VPREB1, TOP3B, PPM1F, MAPK1, YPEL1, PPIL2, ssc-mir-130b, SDF2L1, YDJC, HIC2*
14	119.63 ~ 120.11	*0*
18	53.05 ~ 55.77	*SUGCT, MPLKIP, CDK13, U6, RALA, YAE1, POU6F2, VPS41, ZNF212, ZNF783, ZNF777, KRBA1*

Table S2. Parts of previously reported QTL for exterior, meat and carcass, reproduction, production, health traits.

QTLID	Gene	Trait Name	Chr	Start_Pos(bp)	End_Pos(bp)
		**Exterior**			
125697	*GNRHR*	Gait score (overall)	8	65489707	65489711
126048	*GNRHR*	Gait score (overall)	8	65489707	65489711
125574	*GC*	Hip structure	8	68345253	68345257
125580	*GC*	Rib shape	8	68345253	68345257
125573	*GC*	Hip structure	8	68345408	68345412
125587	*GC*	Rib shape	8	68345408	68345412
126061	*GC*	Front leg conformation	8	68345408	68345412
223540	*SIPA1L1*	Vertebra number	7	94994177	94994181
223541	*RGS6*	Vertebra number	7	95490014	95490018
24244	*NUMB*	Thoracic vertebra number	7	96660859	96660863
		**Meat & Carcass**			
126047	*GNRHR*	Backfat at tenth rib	8	65489707	65489711
10600	*CCKAR*	Backfat at tenth rib	8	65728729	67687532
126060	*GC*	Backfat at tenth rib	8	68345408	68345412
55729	*C7H15orf39*	Obesity index	7	58385471	58385475
194742	*CYP1A2*	Fat androstenone level	7	58726345	58726349
13459	*PKM*	Average glycolytic potential	7	58857679	59191392
13460	*PKM*	Average lactate	7	58857679	59191392
13461	*PKM*	ATP breakdown rate	7	58857679	59191392
13462	*PKM*	pH 24 hr post-mortem (loin)	7	58857679	59191392
13463	*PKM*	PH for Longissmus dorsi	7	58857679	59191392
13464	*PKM*	Drip loss	7	58857679	59191392
13465	*PKM*	Drip loss	7	58857679	59191392
13466	*PKM*	Average glycolytic potential	7	58857679	59191392
13467	*PKM*	Average glycogen	7	58857679	59191392
13468	*PKM*	ATP breakdown rate	7	58857679	59191392
13469	*PKM*	PH for Longissmus dorsi	7	58857679	59191392
13470	*PKM*	PH for Longissmus dorsi	7	58857679	59191392
13471	*PKM*	Meat color L*	7	58857679	59191392
13472	*PKM*	Drip loss	7	58857679	59191392
13473	*PKM*	Average lactate	7	58857679	59191392
258049	*RGS6*	Number of ribs	7	95490014	95490018
2807	*pigs-1*	Diameter of type IIb muscle fibers	4	72950287	101231014
2808	*pigs-1*	Number of muscle fibers per unit area	4	72950287	101231014
2809	*pigs-1*	Diameter of muscle fibers	4	72950287	101231014
2810	*pigs-1*	Diameter of type IIb muscle fibers	4	72950287	101231014
218235	*F5*	Monounsaturated fatty acid to polyunsaturated fatty acid ratio	4	81446369	81479516
218259	*F5*	Monounsaturated fatty acid to polyunsaturated fatty acid ratio	4	81479516	81479520

218213	*CCDC181*	Monounsaturated fatty acid to polyunsaturated fatty acid ratio	4	81565257	81565261
169091	*DPT*	Intramuscular fat content	4	82301412	82301416
216294	*VPS41*	Backfat between 3rd and 4th last ribs	18	55353962	55353966
		**Reproduction**			
643	*GNRHR*	Corpus luteum number	8	67089009	68318702
62168	*ANPEP*	Epididymis weight	7	55363449	55363453
258225	*C7H15orf39*	Semen volume	7	58385471	58385475
258226	*C7H15orf39*	Sperm concentration	7	58385471	58385475
258227	*C7H15orf39*	Sperm motility	7	58385471	58385475
258228	*C7H15orf39*	Sperm abnormality rate	7	58385471	58385475
220870	*CYP1A2*	Age at puberty	7	58784272	58784276
220264	*DPF3*	Teat number	7	96101507	96101507
220265	*DPF3*	Teat number	7	96128652	96128656
211807	*NUMB*	Teat number	7	96632215	96632219
211808	*NUMB*	Teat number	7	96660859	96660863
211809	*NUMB*	Teat number	7	96694362	96694366
211810	*NUMB*	Teat number	7	96727495	96727499
211811	*NUMB*	Teat number	7	96731836	96731840
220246	*NUMB*	Right teat number	7	96731836	96731840
220258	*NUMB*	Teat number	7	96731836	96731840
211812	*NUMB*	Teat number	7	96743523	96743527
211813	*NUMB*	Teat number	7	96786712	96786716
220245	*NUMB*	Right teat number	7	96786712	96786716
220257	*NUMB*	Teat number	7	96786712	96786716
31211	*LIF*	Total number born alive	14	28783341	54617153
31212	*LIF*	Total number born alive	14	28783341	54617153
31213	*LIF*	Total number born alive	14	28783341	54617153
		**Health**			
29651	*LOC100624788*	Mean corpuscular hemoglobin content	8	66655592	66655596
29652	*LOC100624788*	Mean corpuscular volume	8	66655592	66655596
37792	*SFI1*	Platelet count	14	48250501	48250505
37853	*LSMEM2*	Plateletcrit	14	48852097	48852101
		**Production**			
125537	*GNRHR*	Body width	8	65489707	65489711
10590	*CCKAR*	Average daily gain	8	65728729	67687532
10591	*CCKAR*	Daily feed intake	8	65728729	67687532
10592	*CCKAR*	Daily feed intake	8	65728729	67687532
10593	*CCKAR*	Daily feed intake	8	65728729	67687532
10594	*CCKAR*	Days to 110 kg	8	65728729	67687532
10595	*CCKAR*	Days to 110 kg	8	65728729	67687532
10596	*CCKAR*	Average daily gain	8	65728729	67687532
10597	*CCKAR*	Average daily gain	8	65728729	67687532
10598	*CCKAR*	Average daily gain	8	65728729	67687532

10599	*CCKAR*	Average daily gain	8	65728729	67687532
125506	*GC*	Body length	8	68345253	68345257
125533	*GC*	Body width	8	68345253	68345257
125505	*GC*	Body length	8	68345408	68345412
125536	*GC*	Body width	8	68345408	68345412
3091	*LRRK2*	Average daily gain	4	39972760	91937348
140087	*PRKDC*	Feed conversion ratio	4	79757137	79757141
140088	*SELL*	Feed conversion ratio	4	81310009	81310013
1170	*ITGA2*	Body weight	14	47548452	53269523
